# Low-Cost GNSS and PPP-RTK: Investigating the Capabilities of the u-blox ZED-F9P Module

**DOI:** 10.3390/s23136074

**Published:** 2023-07-01

**Authors:** Umberto Robustelli, Matteo Cutugno, Giovanni Pugliano

**Affiliations:** 1 Department of Engineering, University of Naples Parthenope, 80143 Naples, Italy; 2 University of Benevento Giustino Fortunato, 82100 Benevento, Italy; m.cutugno@unifortunato.eu; 3 Department of Civil, Architectural and Environmental Engineering, University of Naples Federico II, 80125 Naples, Italy; giovanni.pugliano@unina.it

**Keywords:** PPP-RTK, mass-market navigation, high-accuracy positioning, low-cost hardware, u-blox ZED-F9P, Point Perfect

## Abstract

GNSS has become ubiquitous in high-precision applications, although the cost of high-end GNSS receivers remains a major obstacle for many applications. Recent advances in GNSS receiver technology have led to the development of low-cost GNSS receivers, making high-precision positioning available to a wider range of users. One such technique for achieving high-precision positioning is Precise Point Positioning-Real Time Kinematic (PPP-RTK). It is a GNSS processing technique that combines the PPP and RTK approaches to provide high-precision positioning in real time without the need for a base station. In this work, we aim to assess the performance of the low-cost u-blox ZED-F9P GNSS module in PPP-RTK mode using the low-cost u-blox ANN-MB antenna. The experiment was designed to investigate both the time it takes the receiver to resolve the phase ambiguity and to determine the positioning accuracies achievable. Results showed that the u-blox ZED-F9P GNSS module could achieve centimeter-level positioning accuracy in about 60 s in PPP-RTK mode. These results make the PPP-RTK technique a good candidate to fulfill the demand for mass-market accurate and robust navigation since uses satellite-based corrections to provide accurate positioning information without the need for a local base station or network. Furthermore, due to its rapid acquisition capabilities and accurate data georeferencing, the technique has the potential to serve as a valuable method to improve the accuracy of the three-S techniques (GIS, remote sensing, and GPS/GNSS).

## 1. Introduction

Presently, common users can exploit signals from the four Global Navigation Satellite Systems (GNSS) [1], delivered on various frequency bands. The availability of off-the-shelf low-cost multi-constellation multi-frequency receivers has increased access to accurate and precise positioning. Generally, more than 20 satellites are above the visible horizon at any time for a receiver in open-sky conditions. This empowers positioning accuracy up to centimeters in real time [2] for low-cost equipment. Before these advancements, to achieve such a level of accuracy, it was essential to employ Real Time Kinematic (RTK) and Network RTK (NRTK) techniques using expensive high-grade GNSS receivers. Currently, achieving centimeter-level positioning is possible even with affordable equipment as there are now low-cost receivers available in the market that have RTK capabilities. Additionally, some commercial data correction services are being implemented, and expanding their coverage and performance continuously, further enhancing the accuracy of these low-cost solutions. Some potential applications can take advantage of the PPP-RTK technique, including low-cost mobile mapping systems [3], unmanned vehicles [4], and remote sensing [5].

This paper aims to investigate the performance in the positioning domain of a low-cost off-the-shelf multi-frequency and multi-constellation GNSS receiver, namely the u-blox ZED-F9P, fed by the low-cost u-blox ANN-MB antenna. Throughout the paper, the accuracy in the positioning domain will be evaluated by computing the difference between the reference true position and the positions obtained by the RTK and the PPP-RTK techniques. Moreover, we will focus on the data collected and the convergence time. The ZED-F9P, manufactured by u-blox, was released on April 2018 and it was the first mass-market multi-band receiver to concurrently use GNSS signals from all four GNSS constellations (GPS, GLONASS, Galileo, and BeiDou) [6]. To comprehensively assess the performance achievable with the ZED-F9P, we collected three sets of data in three different surveys. Nevertheless, to make them comparable, the same experimental conditions (e.g., hardware, software, and satellite geometry) were guaranteed. The first data collection refers to a PPP-RTK survey with consecutive restarts; here the receiver was forced to restart the ambiguity search generating enough samples for statistical analysis; this allows investigation of both convergence times and positioning accuracy. The second data collection refers to the PPP-RTK survey without consecutive restarts, allowing obtaining continuous measurements; throughout this session, the carrier phase ambiguity resolution was maintained; this was useful to collect the data required for multipath analysis. Finally, the third data collection refers to a zero-baseline RTK survey, made with two ZED-F9P receivers in a base-rover configuration, for comparison purposes in terms of both convergence time and positioning accuracy.

The manuscript is organized as follows: Section 2 illustrates an overview of the PPP-RTK and low-cost positioning literature from an application perspective; Section 3 deals with the presentation of the experiments carried out, indicating the hardware, the software, and the positioning techniques considered; Section 4 shows the analysis of the data collected focusing on the analysis of multipath; Section 5 is devoted to explaining the results obtained both in terms of convergence time and positioning performance; lastly, based on the results, some conclusions are drawn in Section 6 where a short outlook on further work is given.

## 2. State-of-the-Art Review of PPP-RTK and Low-Cost Receiver Positioning

In recent years, PPP-RTK has gained significant attention in the field of GNSS positioning, particularly for low-cost applications. PPP-RTK is a hybrid positioning technique that combines the strengths of both PPP and RTK techniques, providing high-precision positioning solutions with low-cost equipment and without the need for a base station. Compared to Precise Point Positioning (PPP), PPP-RTK can improve the convergence time of the positioning solution. The PPP algorithm exploits precise satellite orbits and clocks to enable the single-receiver users to compute their receiver positions with a decimeter or centimeter accuracy ([7]). It is very complex to fix phase ambiguities to their correct integer value because PPP is not a differential technique thus it is not possible to build double-differences to eliminate phase biases originating from satellite and receiver hardware [8].

Starting from the study of Wubbena et al. [9], several researchers have found different ways to fix the un-differenced phase ambiguities to their correct integer values and calculate a fixed PPP solution introducing the PPP-RTK technique. PPP-RTK, also known as PPP with integer ambiguity resolution (PPP-AR) or integer-PPP (IPPP), extends the PPP concept by providing to single-receiver users, in addition to the orbits and clocks, with information about the satellite phase and code biases, and (optionally) atmospheric delays, thus enabling single-receiver ambiguity resolution [10].

PPP-RTK methods differ in the models used, in the corrections applied, and in the estimation strategies employed. A comprehensive description of these models can be found in Teunissen and Khodabandeh [11].

One study applied a single-frequency PPP-RTK approach to u-blox AEK-4T (a low-grade single-frequency receiver). The experiment was conducted with a sampling interval of 30 s. The authors weighted the network corrections by applying the variance matrix. The average time to successfully resolve the phase ambiguities at integers values was 4 min [12].

PPP-RTK corrections can be provided with different strategies, among them single-station PPP-RTK is a special case of PPP-RTK where corrections are computed, instead of a network, by only one single GNSS receiver, as reported in Khodabandeh [7].

Regarding the processing models, Hou et al. [13] propose a multi-frequency (three) phase-only PPP-RTK model excluding the code observables, applied to the Beidou constellation. The time-to-first-fix (TTFF) was three epochs (data rate is 30 Hz) in contrast with 12 epochs achieved with classic Code Plus Phase (CPP) PPP-RTK. The East, North, and Upward Root Mean Square (RMS) errors were equal to 0.62, 0.37, and 2.08 m for the CPP approach and 0.61, 0.37, and 1.64 m for phase-only, respectively.

Another study proposed a PPP-RTK method with the augmentation from a single reference station instead of using a regional Continuously Operating Reference Station (CORS) network. Two PPP-RTK positioning configurations that differ in the reference station start time were applied. When the reference station started estimating the corrections 7200 s before the test, the RMS of the positioning errors in East, North, and Upward directions were 0.005, 0.008, and 0.011 m whereas when the reference station started estimating the corrections only 30 s before, results reached RMS equal to 0.006, 0.006, and 0.016 m. The TTFF were 1 and 8 epochs, respectively [14].

One study reported some preliminary static tests using the u-blox Point Perfect commercial PPP-RTK data correction service with a low-cost receiver based on u-blox ZED-F9P chip. The results of the experiment indicate that a mean horizontal error equal to 0.08 m with a standard deviation of 0.04 m is achievable after 129 s [15].

Another work assessed the quality of low-cost GNSS equipment, again the u-blox ZED-F9P module, for real-time PPP and high-rate dynamic monitoring applications. A horizontal accuracy at the sub-decimeter-level was found along with a decimeter-level vertical accuracy. Regarding the times required to solve the ambiguities, tens of seconds were needed after each cold start imposed [16].

One study proposed a system to apply the PPP-RTK technology to ground-based pseudolite system [17].

Another study proposed an Integrity Monitoring (IM) strategy for multi-constellation PPP-RTK positioning. IM is required for diverse safety-related applications using Intelligent Transport Systems (ITS) where a sub-meter horizontal protection level (HPL) is expected [18].

The first system to implement a satellite-based PPP-RTK augmentation was the Japanese Quasi-Zenith Satellite System (QZSS) through the Centimeter Level Augmentation Service (CLAS). After this, several nations, including South Korea, Australia, Germany, and Denmark, started to experiment with the deployment of PPP-RTK services. Moreover, Galileo developed the High-Accuracy Service (HAS) providing free corrections through the Galileo signal (E6-B) and by terrestrial means (Internet). The theoretical basis underlying Galileo HAS is PPP; as shown by Naciri et al. [19], the combined Galileo and GPS solutions converged below 0.20 m (horizontal) and 0.40 m (vertical) errors within 6 min in static mode and 7.5 min in kinematic mode at the 95th percentile.

Starting from mid-2020, various private companies began to offer commercial data correction services based on PPP-RTK technology [20]. Trimble Inc. deployed CenterPoint RTX, Novatel developed Terrastar-X, and u-blox released Point Perfect service. Lastly, Teria developed the Teriasat service that, at this point, covers all of France’s territory.

As it concerns the use of low-cost GNSS modules, during recent years, several authors investigated the performance achievable with low-cost equipment.

The authors of [21] demonstrated and verified the performance of a low-cost single-frequency GPS receiver, namely the u-blox LEA 4T, in one of the first low-cost RTK experiment ever. During the test, the receiver was connected to the Nippon GPS data service (NGS) NTRIP caster providing 1 Hz measurements of the nearest reference station (6.1 km baseline). According to the results, accuracies reached mean values of 0.03, 0.05, and 0.08 m for the East, North, and Up components.

Another study investigated the contribution of the GLONASS constellation for two mass-market receivers (u-blox LEA EVK-5T and Leica Geosystems NV08-CSM) in order to analyze the NRTK performance founding that mass-market sensors could be a valid alternative to more expensive receivers for a large number of surveying applications; it was also discovered that when employing low-cost hardware, the contribution of the GLONASS constellation to resolve the phase ambiguities was useless, if not dangerous [22].

The authors of [23] tested the single-frequency u-blox 5T receiver performance using the NRTK approach. They demonstrated that 10 min are needed to estimate the ambiguity phase reaching a centimeter-level of precision in 80% of test results, with a baseline less than 10 km. Particular improvements were noted with a baseline shorter than 1 km whereas increasing the duration of the acquisition time did not give any benefit.

Another study tested the u-blox M8T single-frequency multi-constellation receiver fed by Garmin GA38 low-cost multi-constellation antenna considering both single-base RTK and NRTK methodologies for cadastral applications. They reached a centimeter-level accuracy with a baseline shorter than 3 km in RTK and 0.02 m when the NRTK technique is employed [24].

The authors of [25] developed an autonomous driving system using a single-frequency GNSS RTK, namely the u-blox NEO-M8P, for the commercialization of an autonomous driving vehicle. The authors conducted a path-following kinematic test in good sky-view conditions showing that the developed system reached a positional accuracy of 0.006 m with a standard deviation equal to 0.008 m.

The u-blox NEO-M8P was also evaluated by Lu et al. [26] that analyzed the ambiguity fixed-rate and positioning accuracy of single-frequency GPS+BDS data based on the Constrained LAMBDA (CLAMBDA) method with a baseline length constraint. The experiment results showed that the CLAMBDA method can significantly improve the success rate of the GNSS ambiguity resolution. When the ambiguity is fixed correctly, the solution accuracy reached 0.005 m (horizontal plane) and 0.010 m (vertical) in a static test, and 0.010 (horizontal plane) and 0.020 (vertical) m during the dynamic test.

When u-blox deployed the last generation of GNSS receivers, namely the u-blox ZED-F9P, various scientists analyzed and assessed the positioning potentialities; among them, Janos and Kuras [27] evaluated the positioning accuracy exploiting NRTK in different terrain conditions and with different grade antennas. The results indicated that the ZED-F9P receiver equipped with a low-cost patch antenna is only suitable for accurate measurements in open-sky conditions achieving mean errors equal to 0.009 m for the East component, less than 0.001 m for the North component, and 0.027 m for the Upward component. When the u-blox ZED-F9P was used in combination with a geodetic-grade antenna it could even outperform high-grade receivers, indeed the mean errors were 0.007, 0.002, and 0.011 m for East, North, and Upward components, respectively. In the opposite scenario, e.g., an urban canyon, the hardware configuration including the low-cost antenna reached mean errors equal to 0.02, 0.07, and 0.24 m; with the geodetic-grade antenna mean positioning errors increased to 0.06, 0.03, and 0.04 m.

One paper investigated the performance of a u-blox ZED-F9P receiver, connected to a u-blox ANN-MB antenna, during multiple field experiments. In the RTK mode, the ambiguity fixing rate reached 80%, and horizontal accuracy of a few centimeters was achieved during an hour-long session. Similar accuracy was achieved with the PPP if a session was extended to at least 2.5 h. RTK and NRTK measurements achieved a horizontal accuracy better than 0.050 m and a sub-decimeter vertical accuracy. If a base station constituted by a low-cost receiver was employed, the horizontal accuracy degraded by a factor of two, and such a setup may lead to an inaccurate height determination under dynamic surveying conditions [28]. A comprehensive review of the state-of-the-art relative to PPP-RTK technique can be found in [29].

## 3. Materials and Methods

The hardware employed in this experimentation is the u-blox low-cost GNSS ZED-F9P module; it has 184 channels and tracks: the GPS Coarse/Acquisition (C/A) code on L1 band and modernized L2C signal on L2 band; GLONASS open access signals L1OF and L2OF on L1 and L2 bands, respectively; Galileo E1B and E1C code for open service (OS), safety of life (SoL) and commercial service (CS) on E1 band and E5b signal on E5 band; the B1I and B2I Beidou signals; QZSS L1C/A, L1S and L2C signals and L1 C/A SBAS.

This paper considers the u-blox Point Perfect high-precision GNSS augmentation service. Point Perfect data correction service supports the following GNSS signals: GPS L1 C/A, L2P, L2C, L5, GLONASS L1 C/A, L2 C/A, Galileo E1, and E5A/B [30]. For the present study, three different data sets of about two hours were acquired on a reference point whose coordinates are expressed in the ETRF2000 (2008.0). In particular, the first two exploit the PPP-RTK technique (one with forced restarts to investigate the convergence time and the positioning performance and one without restarts to provide continuous observables required for data analysis) and the last makes use of the RTK technique. The purpose of this last data collection was to define a term of comparison. This research is the continuation of the work performed in [15,31,32,33].

**Figure 1:**
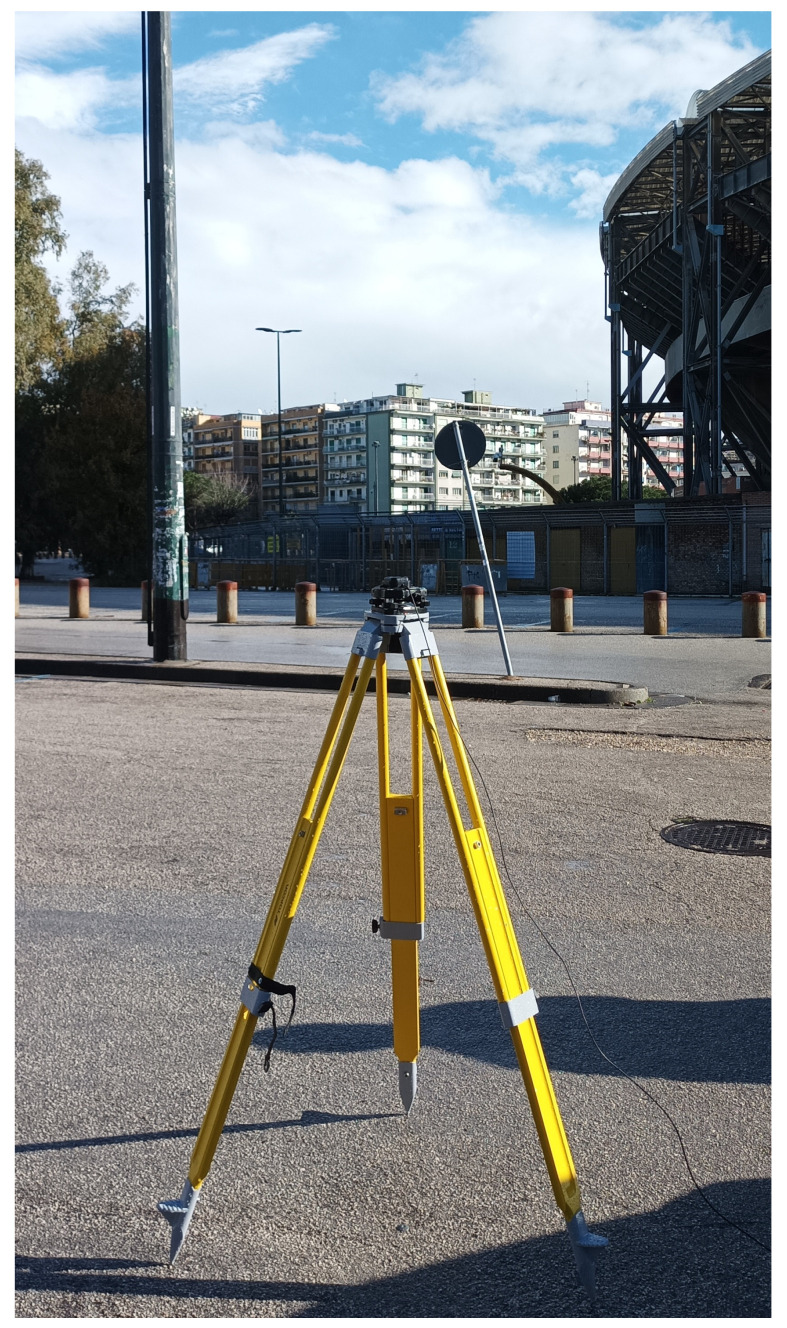
The location where the experimental data were collected.

The data collection site is located in Naples, Italy; this scenario is expected to be a quasi-open-sky and low-multipath environment, as shown in Figure 1. The first data collection session employs a single u-blox ZED-F9P GNSS low-cost receiver connected via SubMiniature version A (SMA) cable to the u-blox ANN-MB low-cost active GNSS antenna [34]. The experiment consisted of a static survey on a point of known coordinates. The data collection refers to a 2.5 h GNSS acquisition in the L1/E1, L2, and L5/E5b bands, carried out on 23rd of December 2022. To guarantee data repeatability, all data collections were interspersed by 861,540 s (about 10 days); hence, the satellite geometry perfectly corresponds [35]. This is verified for all satellites, except for the Galileo E14 and E18 satellites that, due to their very eccentric orbit, exhibit a repeatability of about 20 days [36]. The software employed for data logging and reception of PPP-RTK corrections was the u-blox proprietary evaluation software, known as U-center version 22.07, released on August 2022. Regarding the PPP-RTK data correction service, the commercial service released by u-blox, known as Point Perfect, was evaluated. It can deliver messages both via IP or L-band exploiting the Message Queue Telemetry Transport (MQTT) protocol. Here, the IP network, receiving two types of messages in Secure Position Augmentation for Real-Time Navigation (SPARTN) 2.0 format [37], has been employed: satellite clock corrections every 5 s and satellite orbits, bias, and atmosphere corrections every 30 s. The results were analyzed by exploiting a code written in MATLAB^®^, developed by ourselves. This extracts some information from the binary u-blox proprietary file, e.g., high-precision positions and solution status, among others. During the experiment, the receiver was switched off and restarted (hot start) to simulate signal leakage, going along with a statistical evaluation of the convergence times the system needs to resolve the phase ambiguity at integer values: 60 consecutive hot starts were imposed, interspersed with one minute of fixing maintenance. According to the u-blox integration manual, in hot start mode, the receiver simulates a short-time shutdown (4 h or less), so that its ephemerides are still valid. This strategy leads each time to the receiver starting a new ambiguity search. Given that the Point Perfect data correction service delivers the corrections in ITRF2014 (current epoch), a transformation was performed to consider the relationship of the ETRS89 with the International Terrestrial Reference System (ITRS), allowing the comparison between the ground truth and the PPP-RTK solutions.

The second data collection, conducted on the 2nd of January 2023, still refers to the PPP-RTK technique, but without forcing the receiver to lose the ambiguity. Once the phase ambiguity was fixed at integer values, the acquisition continued to investigate the fixing maintenance capabilities. This also permits obtaining hole-less observations since no restart has been imposed, thus, providing continuous data to conduct a multipath analysis, as reported in Section 4. To assure the same conditions for comparison purposes, the survey was conducted on the same point of known coordinates, employing the same software and collecting the data with the same satellite geometry, as before.

The third data collection session, conducted on the 22nd of January 2023 consisted in a zero-baseline RTK survey where the base station coordinates were expressed in ETRF2000 (2008.0); we employed a couple of u-blox ZED-F9P GNSS low-cost receivers in a base-rover configuration. Both receivers were connected to the same antenna via a splitter.

## 4. Data Analysis

The analysis of satellite geometry, quality of received signals, and multipath error are crucial aspects in the evaluation of GNSS positioning solutions’ accuracy. Throughout the section, the results of these analyses are presented and commented.

**Figure 2:**
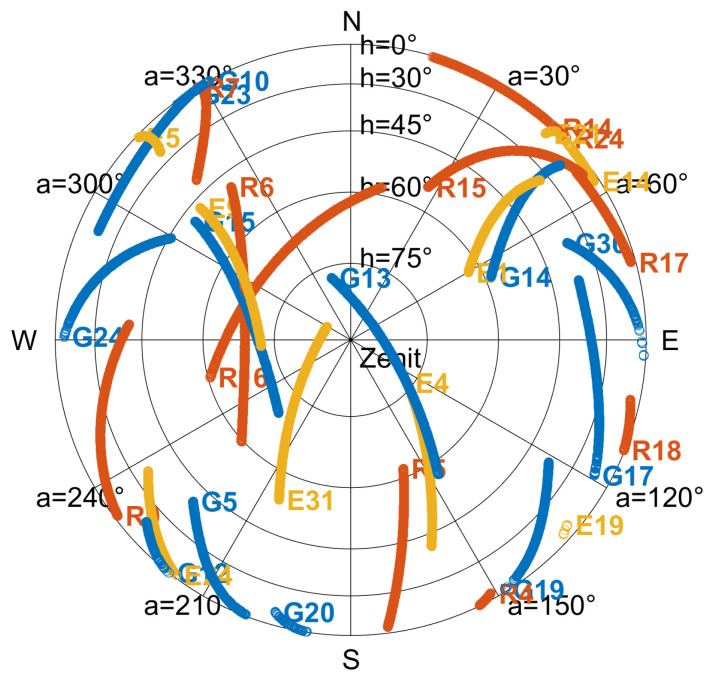
Skyplot of data collected. GPS satellites are represented by blue labels, GLONASS satellites by red labels, and Galileo satellites by yellow ones.

A skyplot of the satellites tracked on the 2nd of January 2023 is shown in Figure 2.

**Figure 3:**
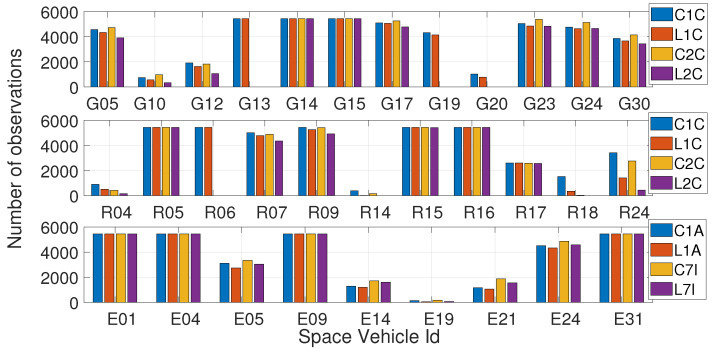
Number of code/phase observables for each tracked satellite. The top panel refers to GPS satellites, the middle panel refers to GLONASS satellites, and the bottom one to Galileo satellites.

The number of observations stored for each satellite on both frequencies has been analyzed, as reported in Figure 3. In particular, the top panel refers to GPS satellites, the middle panel refers to GLONASS satellites, and the bottom one to Galileo satellites. Code and phase observables for the first frequency are represented by blue and red bars, respectively. Code and phase observables for the second frequency are represented by yellow and purple bars, respectively. The figure shows that the receiver was not able to construct the observables on the second frequency for some satellites, namely G13, G19, G20, and R06. Furthermore, for satellites R18 and R24 the number of observables on the second frequency is abnormally low. The GPS satellites listed earlier, belong to the Block IIR launched between 1997 and 2004. These satellites transmit C/A code on the L1 band and precise P(Y) code, both on L1 and L2 bands. Thus, as specified in Section 3, the ZED-F9P GNSS module cannot receive the signals transmitted by GPS Block IIR satellites on the L2 band. Therefore, those could only be acquired with single-frequency observations. During data collection, 12 GPS satellites were in view, 3 of which belong to Block IIR satellites (G13, G19, and G20). To summarize, the receiver managed to track: 12 GPS satellites (9 both on L1 and L2 frequencies), 11 GLONASS satellites (10 both on L1 and L2 frequencies), and 9 Galileo satellites (all of them both on E1 and E5b frequencies).

**Figure 4:**
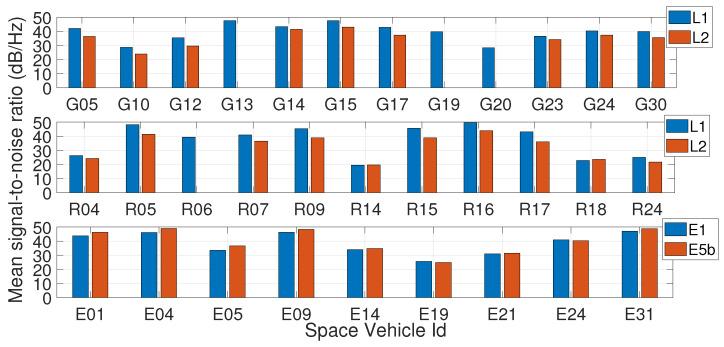
Mean signal-to-noise ratio values for the GPS (**top panel**), GLONASS (**middle panel**), and Galileo (**bottom panel**) satellites. Blue bars refer to the first frequency (L1 for GPS and GLONASS, E1 for Galileo) while red bars refer to the second frequency (L2 for GPS and GLONASS, E5b for Galileo).

Subsequently, the signal-to-noise ratio (SNR) analysis was conducted. Figure 4 reports the mean SNR for GPS (top panel), GLONASS (middle panel) and Galileo (bottom panel) observations acquired by the receiver. Figure 4 suggests that the mean SNR for E1 Galileo frequency is similar to that on E5b, whereas GPS L1 mean SNR is often higher than GPS L2 band. It can be observed that the Galileo E19 satellite’s mean SNR is lower than others; this is explained by considering that this Galileo satellite belongs to the In-Orbit Validation (IOV) Block. Indeed, all IOV satellites were backed-off after the failure of the E20 satellite, which resulted in their signals having less transmitted power than the Full Operational Capability (FOC) satellites.

**Figure 5:**
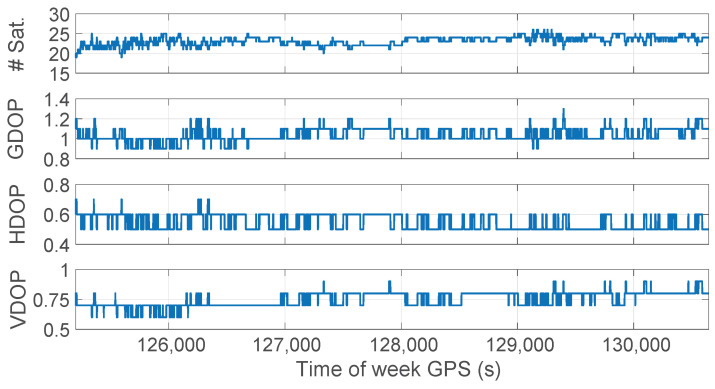
Time evolution of the number of satellites in view (top panel), and dilution of precision parameters (GDOP, HDOP, VDOP) in the other panels, respectively.

The accuracy of the position solution depends on several factors, such as the number and geometry of visible satellites, the accuracy of satellite clocks, and atmospheric conditions. The dilution of precision (DOP) is a quantitative measure of how these factors affect the accuracy of the position solution. Figure 5 depicts the time evolution of the number of satellites in view, the GDOP (Geometric Dilution Of Precision), the HDOP (Horizontal Dilution Of Precision), and the VDOP (Vertical Dilution Of Precision). The figure illustrates that the survey was conducted in excellent conditions for both satellite geometry and horizontal and vertical accuracies.

Another crucial aspect in the analysis of the performance achievable with a low-cost receiver coupled with a low-cost antenna is the multipath error assessment. Therefore, a code multipath analysis on the data collected by the u-blox ZED-F9P GNSS module fed by the u-blox ANN-MB patch antenna has been conducted. To characterize the multipath effect, the analysis exploits the MP observable. It is a linear combination of dual frequencies code and phase measurements. It is used to characterize the magnitude of the multipath and noise contained in the pseudorange observable for any GNSS considered. The availability of dual frequency raw data allowed to assess the multipath performance of the receiver is as follows [38]:

(1) $$mp_{ij}=P_{i}-\left(1+\frac{2}{\alpha -1}\right)\lambda_{i}\phi_{i}+\left(\frac{2}{\alpha -1}\right)\lambda_{j}\phi_{j}=MP_{ij}+\epsilon_{P_{i}}+K+I_{d}$$

(2) $$mp_{ji}=P_{j}-\left(\frac{2\alpha }{\alpha -1}\right)\lambda_{i}\phi_{i}+\left(\frac{2\alpha }{\alpha -1}\right)\lambda_{j}\phi_{j}=MP_{ji}+\epsilon_{P_{j}}+K+I_{d}$$

The variables in Equations (1) and (2) are defined as follows: the subscripts *i* and *j* correspond to the L1 and L2 bands for GPS and GLONASS constellations and E1 and E5b for Galileo constellation, $mp_{ij}$ and $mp_{ji}$ represent the estimated code multipath errors, $P_{i}$ and $P_{j}$ are the code observables, $\lambda_{i}$ and $\lambda_{j}$ are the wavelengths, $\phi_{i}$ and $\phi_{j}$ are the carrier phase observables measured in cycles, $\epsilon_{P_{i}}$ and $\epsilon_{P_{j}}$ are the noise errors in the code measurements, *K* is a constant term related to phase ambiguities, $I_{d}$ is a term related to instrumental delays, and α is defined as the square of the ratio between the *i* and *j* frequencies. The equations contain code multipath errors, noise, and unwanted terms due to phase ambiguities and instrumental delays. To estimate unwanted terms, a moving average filter taken on 900 samples has been employed [33]. The mp observables can only be calculated if code and phase observables are available at two different frequencies. Given that the ZED-F9P GNSS receiver does not receive the second frequency from the IIR Block GPS satellites and the R06 satellite of the GLONASS constellation, multipath for these satellites was estimated using the code-minus-carrier phase (CMC) linear combination (LC) for L1 band. CMC LC is a well-known monitoring metric useful to characterize and measure code multipath errors ([39]) formed with single-frequency data. CMC LC reads as follows

(3) $$P_{r}^{s}-\Phi_{r}^{s}={2I}_{r,i}^{s}-\lambda N_{r,i}^{s}+M_{r,P}^{s}-M_{r,\Phi }^{s}+b_{r,i}-b_{i}^{s}-B_{r,i}+B_{i}^{s}+\epsilon_{r,P}^{s}-\epsilon_{r,\Phi }^{s}$$

The terms in Equation (3) are defined as follows: $\Phi_{r}^{s}$ is the carrier phase observable in meters; $P_{r}^{s}$ is the pseudorange in meters; *s* refers to the satellite and *r* to the receiver; $I_{r,i}^{s}$ is the slant ionospheric delay, in meters; $N_{r,i}^{s}$ refers to the integer ambiguity of phase observations, measured in cycles; $M_{r,\Phi }^{s}$ and $M_{r,P}^{s}$ denote the multipath effect, in the unit of meters, for phase and code observations, respectively; $\lambda_{i}$ is the signal wavelength on selected frequency *i*, measured in meters; $B_{i}^{s}$ and $B_{r,i}$ are the satellite’s and receiver’s phase delays, measured in meters; $b_{i}^{s}$ and $b_{r,i}$ stands for the satellite’s and receiver’s code PR delays, measured in meters, respectively; finally, $\epsilon_{r,P}^{s}$ and $\epsilon_{r,\Phi }^{s}$ are the observed noises of code and phase observations, respectively. CMC LC exposes the noise and multipath of code observations. All other unwanted effects, such as phase ambiguities, satellite and receiver code, and phase biases, which are considered constants, as well as phase multipath and a doubled ionospheric delay, that are time-variant parameters, have to be estimated. These unwanted terms were estimated in the same way as those in the MP combination, e.g., with a moving average filter over 900 samples. The values obtained can be subtracted from the CMCs and MP to obtain the CMC and MP residuals containing only code multipath, noise, and residual error of filtering.

**Figure 6:**
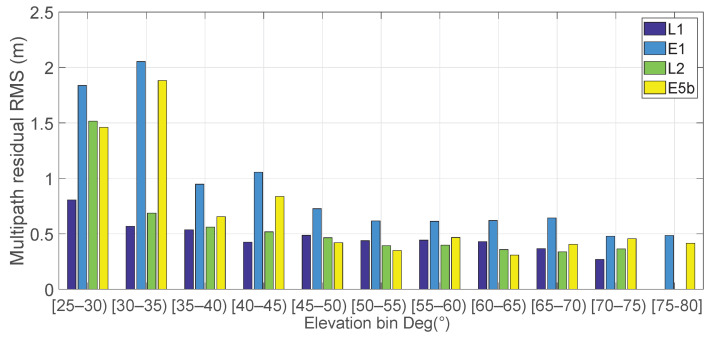
RMS of multipath errors of L1, E1, L2, E5b signals (depicted in blue, light blue, green, and yellow, respectively) as a function of satellite elevation. Each bar represents the RMS of multipath error over elevation bins of 5${}^{\circ }$. The computation multipath’s RMS on L1 and L2 frequencies includes both the GPS and GLONASS systems, while the E1 and E5b frequencies are specific to the Galileo system only.

Figure 6 shows the RMS of multipath error of L1, E1, L2, and E5b signals as a function of satellite elevation. To represent the data in the figure, a mask angle of 25${}^{\circ }$ has been used given that the multipath errors under this mask angle were one order of magnitude higher. It is worth noting that round brackets indicate that the value is not included in the interval whereas square brackets indicate that it is included.

**Figure 7:**
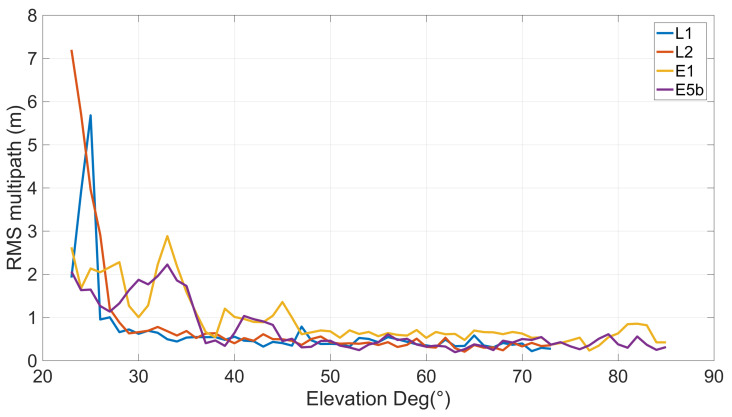
Code multipath on GPS/GLONASS L1 and L2, Galileo E1 and E5b (depicted in blue, orange, yellow, and purple lines, respectively) as a function of satellite elevation. Each point has been calculated as RMS over elevation bins of 1${}^{\circ }$.

Figure 7 shows the code multipath on GPS/GLONASS L1 and L2, Galileo E1 and E5b, as a function of satellite elevation. As expected, as the satellite’s elevation raises the multipath RMS decreases. In particular, multipath RMS for L1 and L2 frequencies are similar and are less than 1 m starting from elevation angles of 26${}^{\circ }$ and maintaining a value of about 0.5 m starting from elevation angles of 50${}^{\circ }$. Conversely, Galileo E1 and E5b multipath RMS present a different behavior: up to 35${}^{\circ }$ they attest to a mean value of about 2 m whereas they maintain a mean value of 0.70 m and 0.50 m starting from 50${}^{\circ }$, respectively.

**Figure 8:**
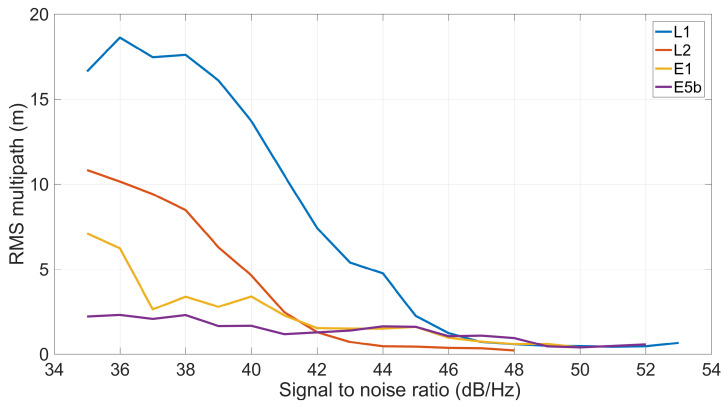
Code multipath on GPS/GLONASS L1 and L2, Galileo E1 and E5b (depicted in blue, orange, yellow, and purple lines, respectively) as a function of SNR. Each multipath value has been calculated as RMS over SNR bins of 1 dB/Hz.

Figure 8 shows the code multipath on GPS/GLONASS L1 and L2, Galileo E1 and E5b, as a function of SNR. The figure illustrates that L1 and L2 frequencies are subjected to the multipath effect as more as the SNR is lower. Conversely, Galileo signals are less affected by this correlation. Furthermore, it can be noticed that the maximum SNR for L2 frequency is equal to 48 dB/Hz.

To summarize, it should be highlighted that the good environmental conditions, both in terms of satellites in view and sources of multipath, resulted in high values of SNR and low code multipath errors.

## 5. Results

The section describes the results of the experiment. The times required to resolve the phase ambiguity both at float and integer values in PPP-RTK mode have been investigated, and then compared with RTK performance. During data collection, 60 hot starts have been imposed; thereby, the receiver has been forced, each time, to search for a new phase ambiguity resolution at integer values. The purpose of the experiment was to assess the receiver’s performance and time needed for reacquiring phase ambiguity resolution after various restarts. This will help determine the receiver’s ability to maintain accurate positioning in challenging environments.

**Figure 9:**
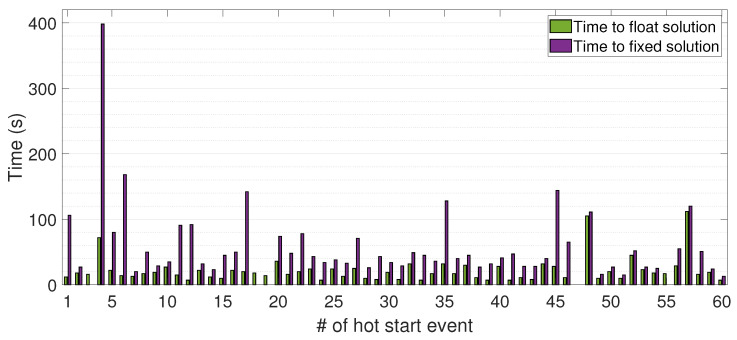
Times to obtain the float and fixed PPP-RTK solutions after each hot start imposed. Times relative to float and fixed solutions are represented by green and purple bars, respectively.

Figure 9 reports the times taken by the low-cost receiver for float and fixed ambiguity estimates after each hot start imposed. During five events (3, 18, 19, 47, and 55), the receiver was unable to resolve the phase ambiguity at integer values; therefore, when this happens, a hot start was imposed after a pre-established time. During event 4, the receiver needed about 400 s to resolve the ambiguity at integer values. Moreover, within event 47, it is worth noting that the receiver was unable to even resolve the phase ambiguity at float values, resulting in the need to restart the software. As far as this is concerned, throughout the experimentation some software malfunctions and bugs came out. The results obtained are outperforming if compared with those achieved by Naciri et al. [19] that applied Galileo HAS correction in static mode obtaining a convergence time of about 6 min.

**Figure 10:**
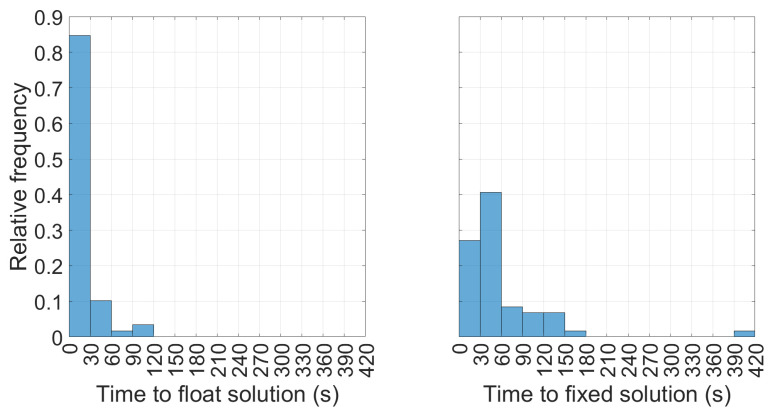
Histograms of times to obtain PPP-RTK float solutions (**left panel**) and times to obtain PPP-RTK fixed solutions (**right panel**).

Figure 10 reports the histograms indicating the relative frequencies of the time to obtain the PPP-RTK float and the fixed solutions. In the (0, 30) s interval the 85% of events reached the float solution with an average time (on all samples) equal to 21.8 s whereas in the (0, 60) s interval the 68% of events reached the fixed solution with an average time (on all events) of 59.4 s.

**Figure 11:**
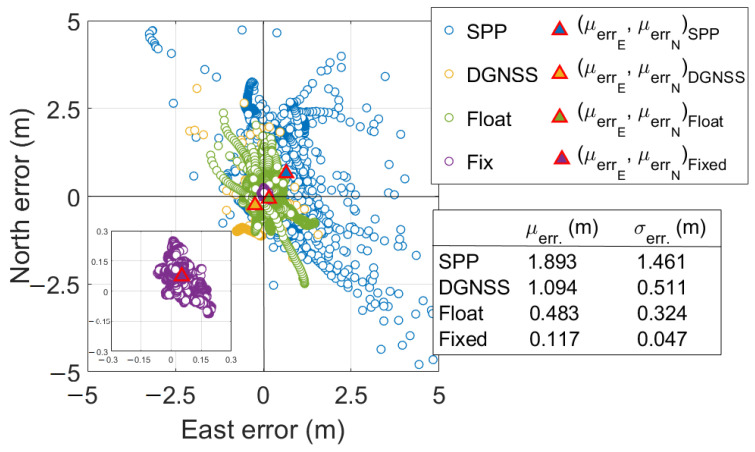
Horizontal coordinate component errors scatter plot of PPP-RTK with forced restarts. Blue, orange, green, and purple markers refer to Single Point Positioning (SPP), Differential GNSS (DGNSS), PPP-RTK float, and PPP-RTK fixed solutions, respectively. Triangles with blue, orange, green, and purple centers refer to SPP, DGNSS, float, and fixed scatter mean points, respectively. The bottom-left of the figure shows a zoomed-in version to improve the readability of fixed solutions. The bottom-right shows a table containing the mean horizontal errors and the corresponding standard deviations for each type of solution status.

Figure 11 shows the scatter plot of horizontal positioning errors of PPP-RTK with forced restarts. The figure reports mean horizontal errors and corresponding standard deviations. It could be noted that mean horizontal errors were equal to 1.893, 1.094, 0.483, and 0.117 m for SPP, DGNSS, PPP-RTK float, and PPP-RTK fixed solutions, respectively; the corresponding standard deviations were equal to 1.461, 0.511, 0.324, and 0.047 m. Inside Figure 11, on bottom-left, has been included a zoomed-in version to provide a closer view of PPP-RTK fixed solutions for better readability; its purpose is to underline that a systematic error of about 0.100 m is present, as confirmed by the mean error statistic.

**Figure 12:**
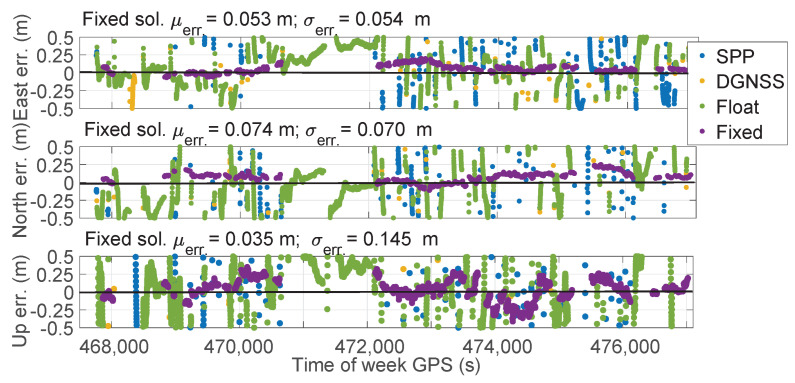
Time series of East, North, and Upward coordinate component errors of PPP-RTK with forced restarts for each type of solution status. Blue markers refer to SPP solutions. Orange markers refer to DGNSS solutions. Green and purple markers refer to PPP-RTK float and fixed solutions, respectively. The top panel indicates the East error. The middle panel depicts the North component while the bottom one refers to the Up component.

Figure 12 shows the East, North, and Upward error components over time for all the types of solutions. The restarts imposed can be clearly identified. Mean errors of fixed solutions, as reported in each panel of Figure 12, were equal to 0.053, 0.074, and 0.035 m for the East, North, and Upward components; the corresponding standard deviations were 0.054, 0.070, and 0.145 m. As specified above, during five events, no fixed solution was achieved; hence, in correspondence of these events, no purple marker is shown in Figure 12.

Table 1 reports the positioning metrics relative to the accuracy and the precision obtained during the PPP-RTK data collection carried out with forced restarts and related to the different types of solution statuses. It should be noted that one solution of the SPP technique was affected by a gross error (orders of kilometers) and to calculate properly the metrics this has been removed. The declared performance standards specified by u-blox state that 95% of horizontal fixed solutions are less than 0.06 m. Regarding the horizontal components, a 2D accuracy (DRMS) of 0.126 m and a precision (95%) of 0.089 m were found; degrading to float solutions the DRMS was equal to 0.583 m, and the 95% to 0.656 m. Moreover, when PPP-RTK corrections were not applied and the system was receiving corrections from EGNOS (DGNSS solution status), the DRMS was equal to 1.207 m and the 95% to 0.114 m. For SPP solutions, DRMS was equal to 2.391 m and 95% to 2.751 m. As expected, the metrics related to the Upward component are worse; an accuracy (RMS) of 0.149 m and a precision (95%) of 0.284 m were found; degrading to float solutions, the RMS was equal to 0.981 m and 95% to 1.905 m. When the solution status is DGNSS type, the RMS was equal to 2.826 m and 95% to 5.456 m. Lastly, the RMS for SPP solutions was 5.219 m and the 95% was 10.13 m. Comparable accuracies have been achieved by Wielgocka et al. [28], though 2.5 h were necessary.

**Figure 13:**
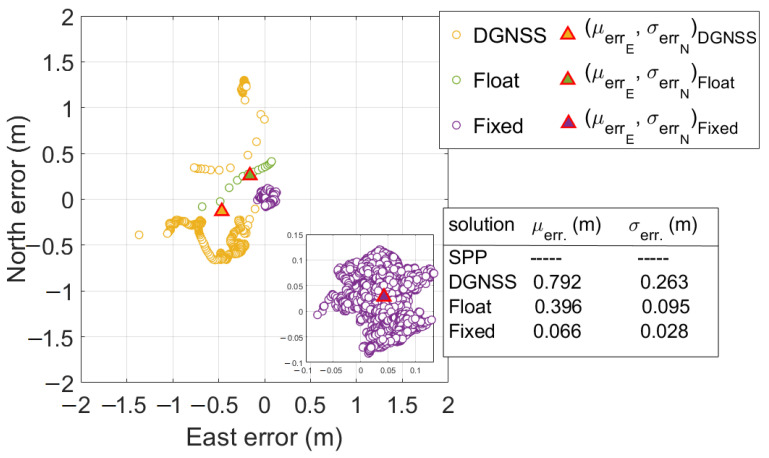
Horizontal coordinate component errors scatter plot for the PPP-RTK without forced restarts. Orange, green, and purple markers refer to DGNSS, PPP-RTK float, and PPP-RTK fixed solutions, respectively. Triangles with orange, green, and purple centers refer to DGNSS, float, and fixed scatter mean points, respectively. The bottom-right of the figure shows a zoomed-in version to improve the readability of fixed solutions. The right part of the figure shows a table containing the mean horizontal errors and the corresponding standard deviations for each type of solution status.

Figure 13 shows the scatter plot of horizontal positioning errors for PPP-RTK without forced restarts. The figure reports mean horizontal errors and corresponding standard deviations. It could be noted that mean horizontal errors were equal to 0.792, 0.396, and 0.066 m for DGNSS, float, and fixed solutions, respectively; the corresponding standard deviations were equal to 0.263, 0.095, and 0.028 m. Inside Figure 13, on the bottom-right, we included a zoomed-in figure to provide a closer view of fixed solutions for better readability; its purpose is to underline that a systematic error of about 0.060 m is present, as confirmed by the mean error statistic.

**Figure 14:**
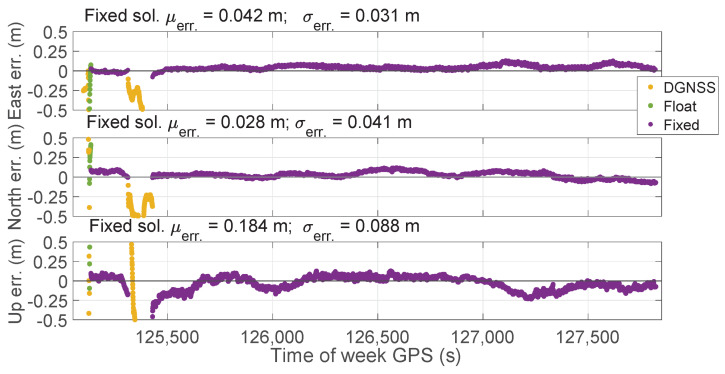
Time series of East, North, and Upward coordinate component errors for the PPP-RTK without forced restarts for all the types of solution statuses. Orange markers refer to DGNSS solutions. Green and purple markers refer to PPP-RTK float and fixed solutions, respectively. The top panel indicates the East error. The middle panel depicts the North component while the bottom one refers to the Up component.

Figure 14 shows the East, North, and Upward error components over time for all the types of solutions for the PPP-RTK without forced restarts. Once the phase ambiguity was fixed at integer values, it was left running for an epoch-by-epoch high-precision position calculation, allowing the assessment of the accuracy in the positioning domain over a long period. Nevertheless, it can be noted that after 426 s the ambiguity at integer values was lost. This happened because the software failed to receive the corrections; hence, it was necessary to configure again the correct parameters in the MQTT client. Once performed, the delivered correction messages were received again and the receiver was able to resolve the ambiguity at integer values maintaining it until the end of the experiment. Mean errors of fixed solutions, as reported in each panel of Figure 14, were equal to 0.042, 0.028, and 0.184 m for the East, North, and Upward components; the corresponding standard deviations were 0.031, 0.041, and 0.088 m. Analyzing Figure 13 and Figure 14, it can be highlighted that SPP solutions are absent whereas DGNSS solutions are mainly grouped in the interval corresponding to the failed corrections delivery event.

Table 2 depicts the positioning performance metrics related to the different types of solution statuses. Given the mode of acquisition, e.g., no restarts, only the statistics related to the solution when the phase ambiguity is fixed at integer values may be emphasized; regarding the horizontal components, fixed solutions showed a 2D accuracy (DRMS) of 0.072 m and a precision (95%) of 0.050 m. Regarding the vertical error statistics, the integer ambiguity fixed solutions revealed an RMS of 0.204 m and a 95% of 0.172 m. To define a term of comparison for the PPP-RTK technique, further data collection exploiting the RTK technique was conducted using two low-cost GNSS u-blox ZED-F9P receivers in a zero-baseline base-rover configuration. It analyzed 66 hot starts to evaluate the receiver’s ability in RTK mode to recover phase ambiguity resolution and maintain accurate positioning.

**Figure 15:**
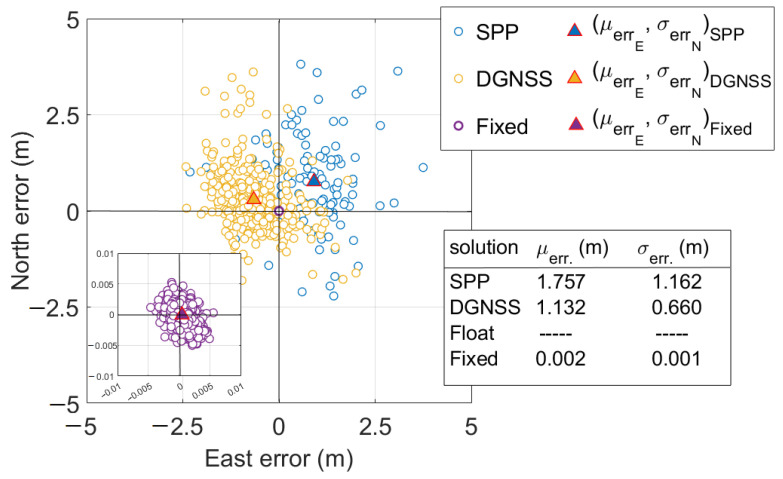
Horizontal coordinate component errors scatter plot. Blue, orange, and purple markers refer to SPP, DGNSS, and PPP-RTK fixed solutions, respectively. Triangles with blue, orange, and purple centers refer to SPP, DGNSS, and fixed scatter mean points, respectively. The bottom-left of the figure shows a zoomed-in version to improve the readability of fixed solutions. The bottom-right shows a table containing the mean horizontal errors and the corresponding standard deviations for each type of solution status.

Figure 15 shows the scatter plot of horizontal positioning errors in RTK mode. The figure reports mean horizontal errors and corresponding standard deviations. It could be noted that mean horizontal errors were equal to 1.757, 1.132, and 0.002 m for SPP, DGNSS, and RTK fixed solutions, respectively; the corresponding standard deviations were equal to 1.162, 0.660, and 0.001 m. Inside Figure 15, on bottom-left, has included a zoomed-in figure to provide a closer view of fixed solutions for a better readability.

**Figure 16:**
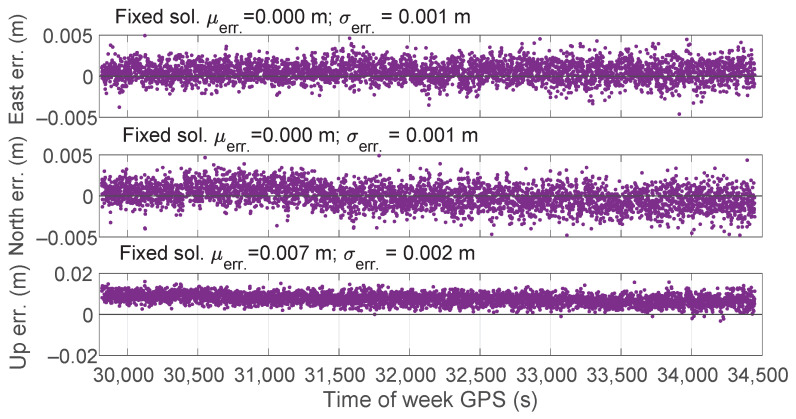
Time series of East, North, and Upward coordinate component errors for the fixed solutions in RTK mode. The top panel indicates the East error. The middle panel depicts the North component while the bottom one refers to the Up component.

Figure 16 shows the East, North, and Upward error components over time in RTK mode for all the types of solutions. Mean errors of RTK fixed solutions (also reported in each panel of the figure) were almost zero for the East and North components ($4\times 10^{-4}$ and $-5\times 10^{-5}$ m, respectively), whereas attested to 0.007 m for the Upward component; the corresponding standard deviations were equal to 0.001, 0.001, and 0.002 m.

Table 3 depicts the positioning performance metrics related to the different solution types in RTK mode. Regarding the horizontal components, RTK fixed solutions showed a 2D accuracy (DRMS) equal to 0.002 m and a precision (95%) equal to 0.002 m. Regarding the vertical error statistics, the integer ambiguity fixed solutions revealed an RMS of 0.008 m and a 95% of 0.005 m. The table is missing the metrics related to float solutions since the receiver always moved directly from DGNSS solutions to fixed ones. DGNSS solutions revealed a DRMS of 1.310 m and a 95% of 1.214 m. DGNSS vertical RMS was equal to 2.531 m and 95% was equal to 3.695 m. Lastly, as it concerns the SPP solution, the DRMS was equal to 2.104 m and the 95% of 2.036 m whereas the vertical RMS is 3.531 m and the 95% was 5.990 m.

**Figure 17:**
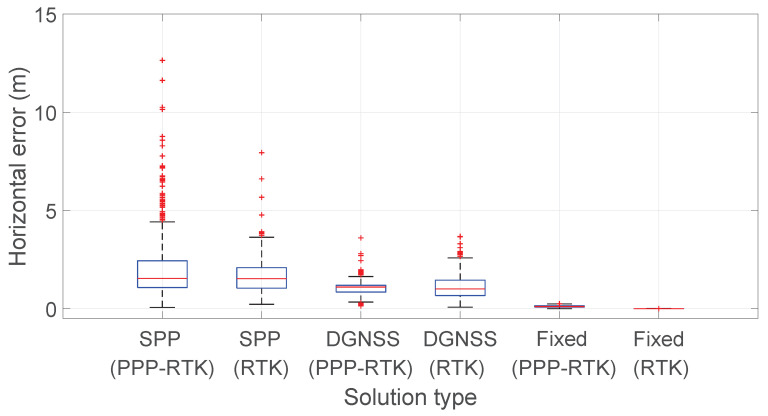
Comparison of Horizontal error box-plots for PPP-RTK with forced restarts and RTK considering SPP, DGNSS, and fixed solutions statuses.

Figure 17 illustrates the horizontal error box-plots comparison for SPP, DGNSS, and fixed solutions statuses considering both PPP-RTK with forced restarts and RTK modes. The SPP boxes of both experiments considered have about the same size whereas PPP-RTK has better performance when DGNSS solutions are achieved. Moreover, it is worth noting that SPP solutions of PPP-RTK present more outliers than SPP of RTK. Lastly, the RTK fixed solutions box is more compact than that of PPP-RTK fixed, solution. The comparison described above should take into account the number of samples; indeed, the SPP PPP-RTK box-plot has been calculated over 1243 samples while the SPP RTK box-plot over 125. As it concerns the number of samples of DGNSS and fixed solutions, these are comparable between PPP-RTK and RTK experiment, as shown in Table 1 and Table 3. Furthermore, the improvement of horizontal accuracy switching from SPP to DGNSS, then to PPP-RTK float, and finally to PPP-RTK fixed solutions is evident. The box related to phase ambiguity fixed solutions at integer values is more compact than the one related to the phase ambiguity resolved at float values which in turn is more compact than the one related to the DGNSS solutions and SPP. This type of representation allowed the highlighting of the level of accuracy achieved when the PPP-RTK fixed solution is available with respect to the other solutions even in relation to outliers. This behavior repeats for the RTK experiment, e.g., moving from SPP to RTK fixed solutions, the boxes gradually become more compact and shifted downwards. The representation also indicates that when the phase ambiguity is resolved at integer values, no more outliers are present.

## 6. Conclusions

In this work, we investigated the performance of the u-blox ZED-F9P GNSS low-cost module for static PPP-RTK. Furthermore, a in-depth analysis of the data collected was carried out in Section 4.

The results obtained from the analysis of the collected data demonstrate that PPP-RTK has proven to be a promising technique for centimeter-level positioning performance with low-cost receivers. During the experiments, the time-to-fix was investigated after consecutive receiver shutdowns.

The PPP-RTK acquisition with forced restarts showed that the employment of cost-effective equipment, along with the exploitation of commercial PPP-RTK data correction service, allowed reaching submeter-level accuracy in about 20 s and centimeter-level accuracy in about 60 s once the integer ambiguity is fixed. In particular, the receiver 2D accuracy (DRMS) was equal to 0.126 m and the precision (95%) was 0.089 m. Regarding vertical positioning, the phase ambiguity fixed solutions revealed an RMS equal to 0.149 m and a 95% of 0.284 m. The positioning performance metrics for solutions with phase ambiguity at float values reached a DRMS of 0.583 m and a 95% of 0.656 m while the RMS for vertical positioning was equal to 0.981 m and the 95% was 1.905 m.

The analysis of the data collected in the zero-baseline RTK configuration revealed that classical RTK is still the best-performing positioning technique both in terms of convergence times and positioning accuracy. Indeed, the experiment revealed a DRMS of 0.002 m and 0.008 m for horizontal and vertical components, respectively; the corresponding 95% was equal to 0.002 m and 0.005 m. The receiver in RTK configuration outperforms also for what concerns the time to converge, since an average time of 8 s was found. Even if the RTK surmounts the PPP-RTK both in terms of convergence times and positioning performance, it must be taken into account that the application of RTK to mass-market mobility is unfeasible for various factors, among all: the need for expensive ground infrastructures and limited bandwidth capabilities. These reasons make the PPP-RTK technique a good candidate to fulfill the demand for mass-market accurate and robust navigation since uses satellite-based corrections to provide accurate positioning without the need for a local base station. PPP-RTK overcomes the limitations of RTK using precise clock and orbit data from a scaled network of reference stations to calculate accurate corrections for the mobile receiver, making it a more practical solution for mass-market mobility applications. Despite its advantages, the PPP-RTK technique still faces some limitations, e.g., the need for a stable and reliable network connection when receiving data via IP. Nonetheless, the ongoing advancements in GNSS receiver technology and data communication networks address these limitations and enable PPP-RTK to become a viable and cost-effective option for high-precision positioning applications.

An interesting future development for this research will be testing the system during kinematic experiments since the demand for ubiquitous positioning, mainly related to vehicle navigation applications, is growing on a continuous basis. Furthermore, the low-cost GNSS module performances can be improved by integrating a low-cost IMU, to overcome the problems related to signal interruptions, hence improving the robustness of the integrated system.

## Figures and Tables

**Table 1:** Positioning performances of the PPP-RTK with forced restarts for the different types of solution statuses.

Solution Type	# of Solutions	Horizontal		Vertical	
		DRMS (m)	95% (m)	RMS (m)	95% (m)
SPP	1243	2.391	2.751	5.219	10.13
DGNSS	160	1.207	1.114	2.826	5.456
Float	3930	0.583	0.656	0.981	1.905
Fixed	3618	0.126	0.089	0.149	0.284

**Table 2:** Positioning performance for the different types of solution statuses. The table refers to the PPP-RTK without forced restarts.

Solution Type	# of Solutions	Horizontal		Vertical	
		DRMS (m)	95% (m)	RMS (m)	95% (m)
SPP	0	-	-	-	
DGNSS	290	0.835	0.720	1.642	2.856
Float	14	0.406	0.308	0.877	0.522
Fixed	5116	0.072	0.050	0.204	0.172

**Table 3:** Positioning performance for the different types of solution statuses in RTK mode.

Solution Type	# of Solutions	Horizontal		Vertical	
		DRMS (m)	95% (m)	RMS (m)	95% (m)
SPP	125	2.104	2.036	3.561	5.990
DGNSS	332	1.310	1.214	2.531	3.695
Float	2	-	-	-	-
Fixed	4136	0.002	0.002	0.008	0.005

## Data Availability

All relevant data are included in the manuscript.

## Abbreviations

The following abbreviations are used in this manuscript:

CLAMBDA	Constrained Least-squares AMBiguity Decorrelation Adjustment
CLAS	Centimeter Level Augmentation Service
CMC-LC	Code Minus Carrier-Linear Combination
CORS	Continuously Operating Reference Station
CPP	Code Plus Phase
CS	Commercial Service
DGNSS	Differential GNSS
DOP	Dilution Of Precision
EGNOS	European Geostationary Navigation Overlay System
FOC	Full Operational Capability
GDOP	Geometric Dilution Of Precision
GNSS	Global Navigation Satellite System
HAS	High-Accuracy Service
HDOP	Horizontal Dilution Of Precision
HPL	Horizontal Protection Level
IM	Integrity Monitoring
IMU	Inertial Measurement Unit
IOV	In-Orbit Validation
IPPP	Integer Precise Point Positioning
ITS	Intelligent Transport System
MP	Multipath
MQTT	Message Queue Telemetry Transport
NGS	Nippon GPS data Service
OS	Open Service
PPK	Post-Processed Kinematic
PPP	Precise Point Positioning
PPP-AR	Precise Point Positioning-Ambiguity Resolution
PPP-RTK	Precise Point Positioning-Real Time Kinematic
QZSS	Quasi-Zenith Satellite System
RMS	Root Mean Square
RTK	Real Time Kinematic
SoL	Safety of Life
SMA	SubMiniature version A
SPAN	Synchronized Position, Attitude and Navigation
SPARTN	Secure Position Augmentation for Real Time Navigation
SPP	Single Point Positioning
TTFF	Time-To-First-Fix
VDOP	Vertical Dilution Of Precision
